# Reply to a Letter to the Editor on *Comparative performance of fully-automated and semi-automated artificial intelligence methods for the detection of clinically significant prostate cancer on MRI: a systematic review*

**DOI:** 10.1186/s13244-023-01594-4

**Published:** 2024-02-14

**Authors:** Nikita Sushentsev, Tristan Barrett, Leonardo Rundo

**Affiliations:** 1 Department of Radiology, Addenbrooke’s Hospital and University of Cambridge, Cambridge, UK; 2 Department of Information and Electrical Engineering and Applied Mathematics (DIEM), University of Salerno, Fisciano, SA Italy

**Keywords:** Artificial intelligence, Machine learning, Prostate cancer

Dear Editor-in-Chief,

We have read the letter concerning our recent publication in *Insights into Imaging* titled *Comparative performance of fully-automated and semi-automated artificial intelligence methods for the detection of clinically significant prostate cancer on MRI: a systematic review* [1] on our previously published work [2].

First, we acknowledge that some of the concerns raised by the authors are valid, including the points raised about specific numbers in both columns in Table 4 of the originally published article, as well as columns “PPV” and “Threshold” in Table 5. While important to highlight, these oversights do not change any of the review conclusions.

That said, we strongly disagree with the authors on other points mentioned in their letter. First, the point raised regarding Table 1 of the originally published article only supports our decision to assign a “high risk of bias” to this study. Specifically, the authors confirm that their study “considers as the clinical standard the TRUS biopsy performed 6 weeks before MRI.” This approach contradicts all major European, American, and British guidelines that specify pre-biopsy (not post-biopsy) MRI as the first-line diagnostic tool in patients with suspected prostate cancer. In addition, performing MRI only 6 weeks after biopsy is likely to yield the presence of residual hemorrhage, which can severely skew image-derived radiomic features, many of which relate to microstructural tissue properties. With that in mind, our background document only allows at least a 6-month period between biopsy and MRI to allow for the restoration of the unperturbed tissue architecture. Second, the authors provide their own interpretation of the QUADAS-2 Background document that suits their clinical protocol, which represents a biased approach in its own right. We, therefore, do not consider this concern as an error.

Moreover, in the point related to the column “Threshold” in Table 5 of the originally published article, the authors speculate about values derived from other studies, particularly questioning the “negative values” that are provided in this column. This refers to a specific study that does indeed report a negative value for the selected threshold, which again does not constitute an error on our part.

Finally, in the point regarding the columns “Accuracy” and “NPV” in Table 5 of the originally published article, the authors themselves confirm that they did not explicitly report accuracy and negative predictive value. Naturally, in our review, this was recorded as “NR.” Calculating any performance characteristics on behalf of the authors was not within the scope of our work, which again does not constitute an error.

## Data Availability

All information is included in the Reply to the Letter to the Editor.
